# Satisfactory result of great saphenous vein endovenous laser ablation until below the knee on active venous leg ulcer: a case series

**DOI:** 10.12688/f1000research.131695.4

**Published:** 2024-09-20

**Authors:** Taofan Taofan, Iwan Dakota, Achmad Hafiedz Azis Kartamihardja, Jonathan Edbert Afandy, Suci Indriani, Suko Adiarto

**Affiliations:** 1 Department of Cardiology and Vascular Medicine, Faculty of Medicine University of Indonesia / National Cardiovascular Center Harapan Kita / University of Indonesia Academic Hospital, Jakarta, Indonesia; 2 Departement of Cardiology and Vascular Medicine, Faculty of Medicine Padjadjaran University / Hasan Sadikin General Hospital, Bandung, Indonesia; 3 Assistant of Vascular Division, Department of Cardiology and Vascular Medicine, Faculty of Medicine University of Indonesia / National Cardiovascular Center Harapan Kita / University of Indonesia Academic Hospital, Jakarta, Indonesia

**Keywords:** venous leg ulcer, endovenous laser ablation, below the knee, laser power, outcome, complication

## Abstract

**Background:**

Active venous leg ulcer (VLU) is the most severe manifestation of chronic venous disease which not only affects patients’ health, but also decreases the quality of life, and delivers economic burdens. Treatment of superficial venous reflux with early endovenous laser ablation (EVLA) has been associated with reducing ulcer recurrence levels and promoting faster VLU healing. We reported three cases of patients with active VLU undergoing EVLA with different approaches.

**Case illustration:**

Three patients came with complaint of leg ulcer, diagnosed with C6sEpAsdPr, with venous clinical severity scores (VCSS) of 15, 23, and 22 respectively. Severe great saphenous veins (GSV) reflux was found in all patients by duplex ultrasound examination. The second patient had undergone above-the-knee EVLA. All patients underwent EVLA using 1470-nano meter wavelength laser device and ELVeS radial fiber (Biolitec, Bonn, Germany). The laser energy protocol used was 6 W linear endovenous energy density (LEED) 50 J/cm for proximal until media ATK GSV ablation, 5 W LEED 40 J/cm for media ATK until proximal below-the-knee (BTK) GSV, and 2 W LEED 20 J/cm for proximal until distal BTK GSV. The third patient was also treated with EVLA for small saphenous vein severe reflux. Follow-up until 6 months post-EVLA showed significant healing of the ulcer with 14, 16, and 17 VCSS reduction consecutively without any complication.

**Conclusion:**

We’ve reported three cases of patients with active VLU undergoing EVLA until BTK with significant results. The EVLA of GSV until BTK where there is still significant reflux is safe and provides satisfactory results in patients with VLU.

## Introduction

Chronic venous disease (CVD) is defined as a long-term morphological and functional abnormality of the venous system that manifests as symptoms or signs requiring treatment or examination. ^1^ Chronic venous insufficiency (CVI), which correlates with clinical, etiological, anatomical, and pathophysiological (CEAP) classification as C3 through C6, is reserved for advanced CVD and applied to functional anomalies of the venous system that result in edema, skin changes, or venous ulcers. ^1^ Active venous leg ulcer (VLU) is the most severe manifestation of CVD, with several contributing factors including venous reflux, obstruction outflow, pump failure of calf muscles, and obesity that causes chronic venous hypertension. ^1^ The prevalence ranges between 0.8 to 2.2 patients for every 1000 people/year and with 19.0±12.1 weeks healing time. ^2^ Active VLU alone represents 3.5% of patient populations with CVD. ^3^ Despite the small number, the quality of life of patients with active VLU is affected in various dimensions, especially on the emotional level and regarding body image, with pain and ulcer severity contributing to a decrease in patients’ quality of life. ^4^ VLU not only impacts patients’ health but also delivers an economic burden with approximately £7706 per patient per year direct cost to manage VLU patients, and more than £2 billion annually (representing 1.2% of the annual budget) in a study on the British population. ^5^ Patients with an advanced form of chronic venous insufficiency (CVI), including lipodermatosclerosis, healed VLU, and active VLU have a complex venous disease with a superficial, perforator, and deep vein involvement. ^6^ Treatment of superficial venous reflux with early endovenous laser ablation (EVLA) has been associated with reducing ulcer recurrence levels and promoting faster VLU healing. ^7^ In a study by Montminy *et al*., ^8^ the VLU healing rate after 12 months in the patient group treated with EVLA was 82% versus 36% in the compression therapy-only group. Here we report three cases of patients with active VLU undergoing EVLA using a 1470-nano meter (nm) wavelength laser device and ELVeS radial fiber (Biolitec, Bonn, Germany) in the National Cardiovascular Center Harapan Kita, Jakarta, Indonesia with different approaches.

## Case illustration

### Case 1

**Figure 1.**
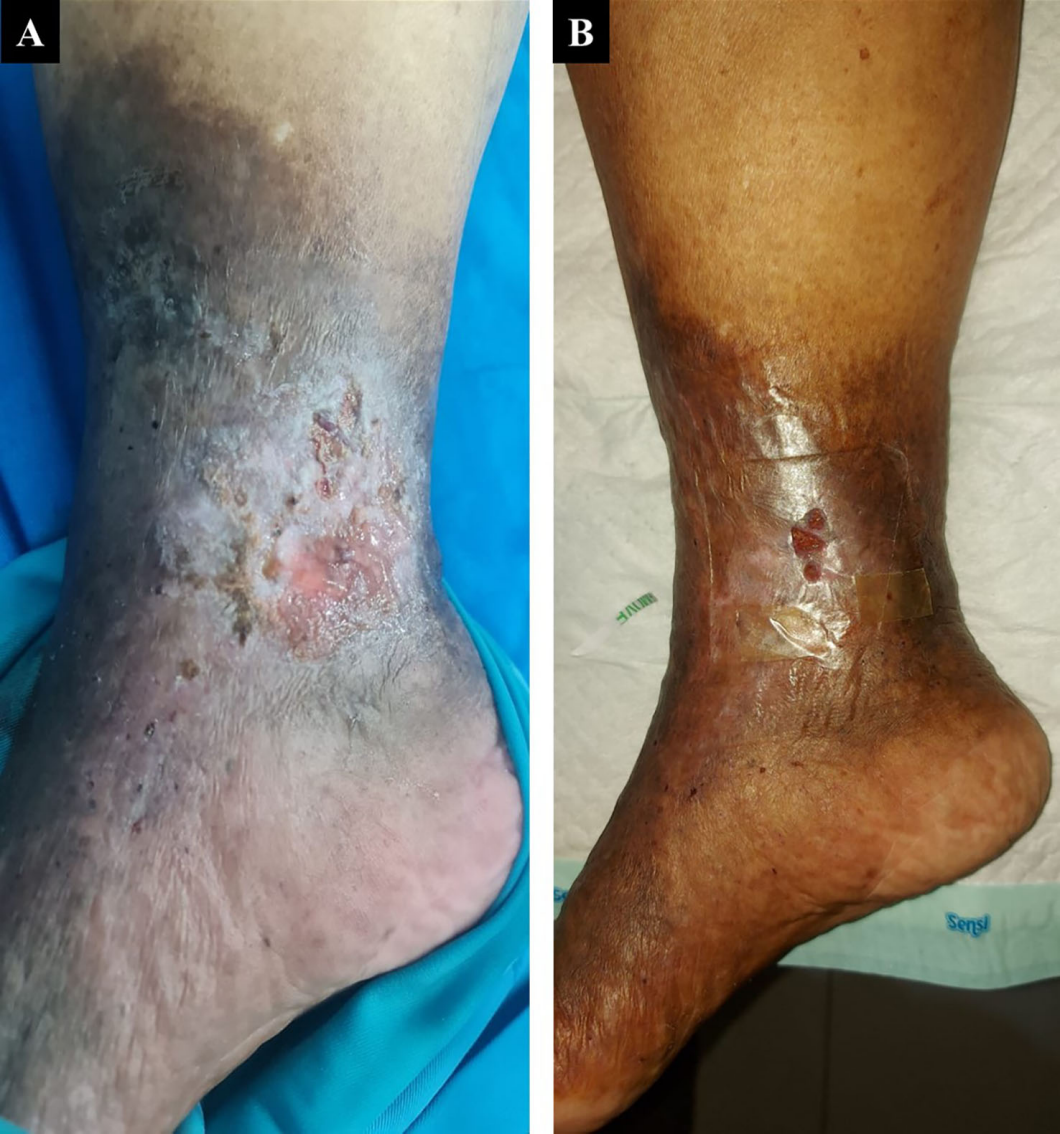
The 1 ^st^ patient rapid progression of venous ulcer healing. A. pre-EVLA; B. 1-week post-EVLA.

A 60-year-old woman, who worked as a baker, presented with a complaint of a wound in her right ankle accompanied by pain (visual analog scale (VAS) 3/10) in the past three months. The complaint began with swelling in both lower limbs that got worse one year earlier. There was a change of skin color on both the patient’s leg and foot which became darker over time. The patient had a history of hypertension. On physical examination of the medial side of the right ankle region, there was a solitary ulcer, sized 3×4 cm with irregular edges, erythema ulcer base accompanied by purulent discharge, dry distant tissue, edema (+), and hyperpigmentation on 1/3 mid of the lower leg until dorsal side of the foot ( Figure 1A). Plethysmography examination revealed her ankle and arm systolic pressure, then it was divided to get the ankle-brachial index (ABI) with a result of her left ABI was 1.25 and her right ABI was 1.06. There was severe reflux (duration >1000 ms) in above the knee (ATK) and below the knee (BTK) great saphenous vein (GSV) and moderate reflux (duration 500 ms – 1000 ms) in the common femoral vein and popliteal vein of both lower limbs from duplex ultrasound (DUS) examination of the lower extremity. No obstruction or thrombus was found in the deep vein. The flow of arteries in her both lower extremities was normal. The patient was diagnosed with CVI on both lower limbs with active VLU on medial side of the right ankle (C6sEpAsdPr). The venous clinical severity score (VCSS) was 15. The patient agreed to EVLA treatment on the right lower limb and received ceftriaxone antibiotics for five days. The procedure was done after the patient finished the antibiotics course.

EVLA was performed under spinal anesthesia. Mapping was done on the right GSV. The diameter of the saphena-femoral junction (SFJ) was 11.0 mm, proximal ATK GSV 9.2 mm, medial ATK GSV 6.0 mm, distal ATK GSV ATK 6.1 mm, proximal GSV BTK GSV 5.2 mm, media BTK GSV 4.5 mm, and distal BTK GSV 3.0 mm. Double puncture was used on distal BTK and distal ATK GSV because of tortuosity and branching at distal ATK. Puncture and application of tumescent anesthesia were done by guided DUS. EVLA started 3 cm from SFJ with laser power of 6 W linear energy density (LEED) 50 J/cm at proximal until media ATK, 5 W LEED 40 J/cm at media ATK until proximal BTK, and 2W LEED 20 J/cm at proximal until distal BTK. The total ablated GSV length was 60 cm with a total of 850 mL tumescent. Lower extremity DUS on the next day showed perfectly obliterated ablated GSV, no thrombus found in the deep vein, and a patent epigastric vein. The patient was discharged one day after the procedure.

The patient’s condition was assessed one week after EVLA, and she reported no more pain, significant rapid improvement of the ulcer, granulation tissue (+), and no more discharge ( Figure 1B). At follow-up until six months post-EVLA, the patient didn’t have any complaints and had a healed ulcer. No recanalization of the treated segment was found by DUS examination. There was a 14-point improvement in VCSS score down to a score of 1.

### Case 2

**Figure 2.**
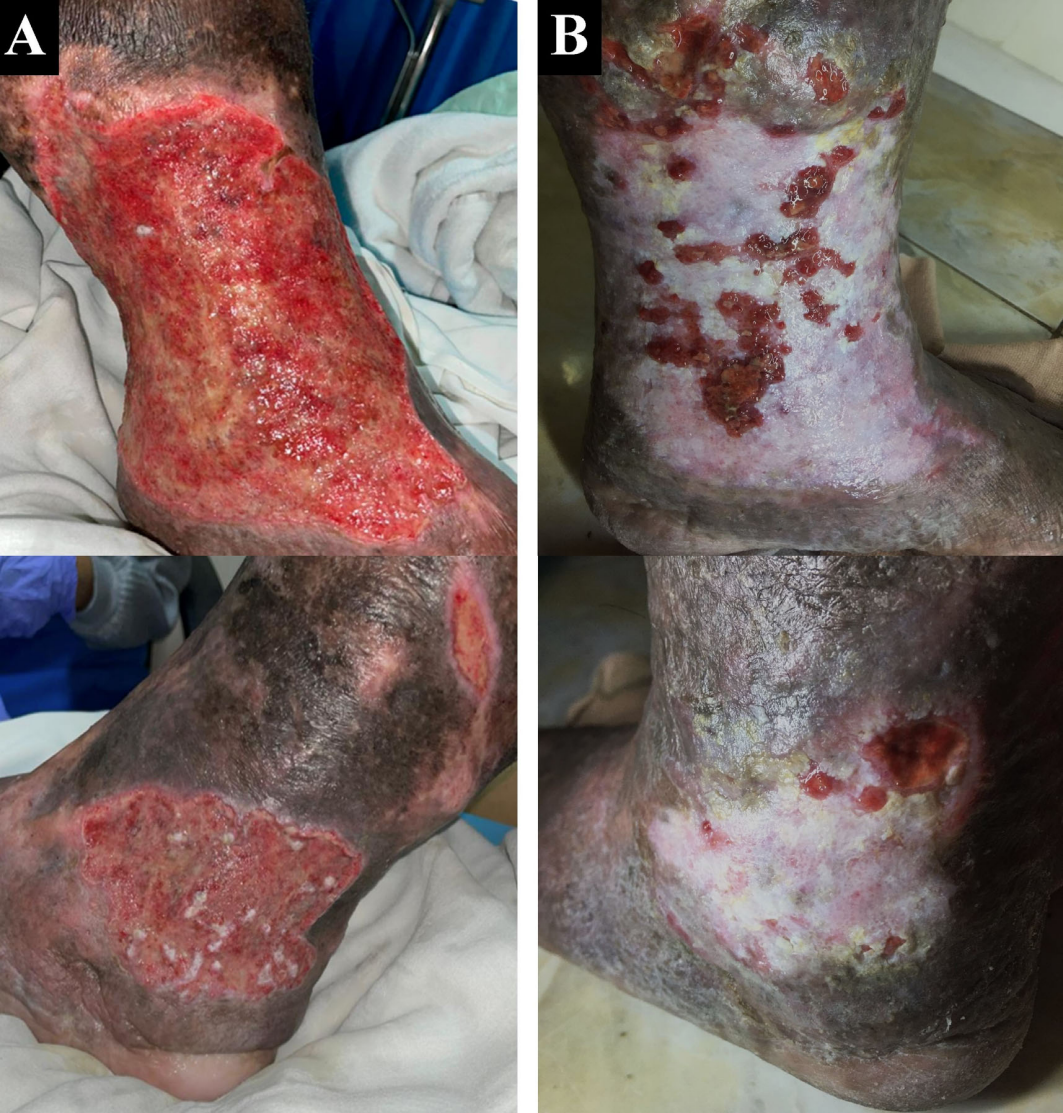
Venous leg ulcer healing progression of the 2 ^nd^ patient. A. pre-EVLA; B. 6-months post EVLA.

**Figure 3.**
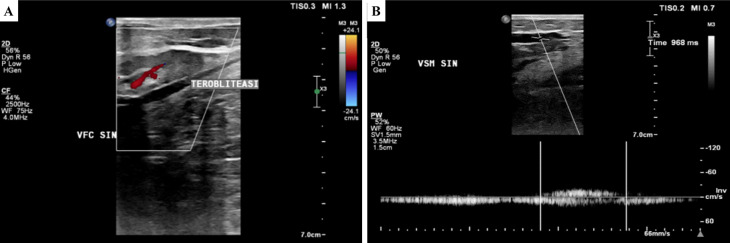
Lower extremity duplex ultrasound of the 2 ^nd^ patient. A. Obliterated segment of above-the-knee left GSV; B. persistent reflux of below-the-knee GSV (968 ms).

A 50-year-old male chef presented with complaints of wounds on his left ankle in the last two years accompanied by pain aggravated by walking (VAS 4/10). The patient had undergone EVLA on the left ATK GSV with phlebectomy eight months earlier, but there wasn’t any improvement of the ulcer. The patient had wound care with a surgeon who suggested doing a lower extremity vessel evaluation. The patient had a history of hypertension, type 2 diabetes mellitus, and obesity with body mass index (BMI) of 39.45 kg/m ^2^. Physical examination of the left ankle region showed multiple ulcers sized 18×10 cm on the medial side, 8×5 cm, and 4×2 cm on the lateral side with irregular edges ( Figure 2A). The ulcer had an erythema base, purulent discharge, dry distant tissue, and edema (+). Hyperpigmentation of 1/3 mid of the left lower leg until dorsal side of the left foot. Plethysmography examination revealed his ankle and arm systolic pressure, then it was divided to get the ABI with a result of his left ABI was 1.14 and his right ABI was 1.10. Lower extremity DUS revealed perfectly obliterated proximal until distal ATK left GSV, severe reflux (duration >1000 ms) on BTK left GSV, common femoral vein, popliteal vein, and accessory veins of both lower limbs, and moderate reflux (duration 500 ms – 1000 ms) in right GSV ( Figure 3). No obstruction or thrombus was found in the deep vein. The flow of arteries in his both lower extremities was normal. The patient was diagnosed with CVI on both lower limbs and active VLU on medial and lateral side of the left ankle region (C6sEpAsdPr) with VCSS 23. The patient decided to undergo EVLA on the left lower limb. Previous results from culture of the ulcer base swab showed *Pseudomonas aeruginosa* (which is resistant to cefazoline). The patient received ceftriaxone and metronidazole for five days. The procedure was done after the patient finished the antibiotics course.

EVLA was done under spinal anesthesia. Mapping of left GSV and accessory vein was done resulting in proximal ATK accessory vein diameter 6.0 mm, medial ATK 8.3 mm, and distal ATK 6.5 mm. The diameter of distal ATK GSV was 8.00 mm, proximal BTK GSV 7.4 mm, and media BTK GSV 5.4 mm. The puncture was initially started from distal ATK GSV retrogradely, but the laser catheter couldn’t pass because of branching in the proximal BTK area. Access was moved to the left dorsalis pedis, and a laser catheter was introduced until distal ATK. The total tumescent amount was 350 mL. EVLA was started in distal ATK GSV until proximal BTK with laser power of 5 W LEED 40 J/cm, followed by 2 W LEED 20 J/cm in proximal until media BTK GSV. The second puncture was done on distal ATK accessory veins with laser power 6 W LEED 50 J/cm from proximal until distal ATK of the left accessory vein. The total amount of the tumescent was 500 mL. The total ablated GSV length was 33 cm and the accessory vein was 29 cm. Lower extremity DUS on the next day showed ATK until BTK left GSV and accessory vein were perfectly obliterated, no DVT found, and patent epigastric vein.

The patient was discharged two days after the procedure with optimal medical treatment to control his risk factor and wound care by the nursing team that follows the tissue, infection/inflammation, moisture, and edge of wound (TIME) concept with modern wound dressing. At follow-up until six months, the patient did not have any complaints. Skin condition on the left ankle region showed healed ulcers with granulation tissues, no purulent discharge, minimal swelling until below the knee, and hyperpigmentation ( Figure 2B). No recanalization of the treated segment was found by DUS examination. There was a 16-point reduction of VCSS down to 7.

### Case 3

**Figure 4.**
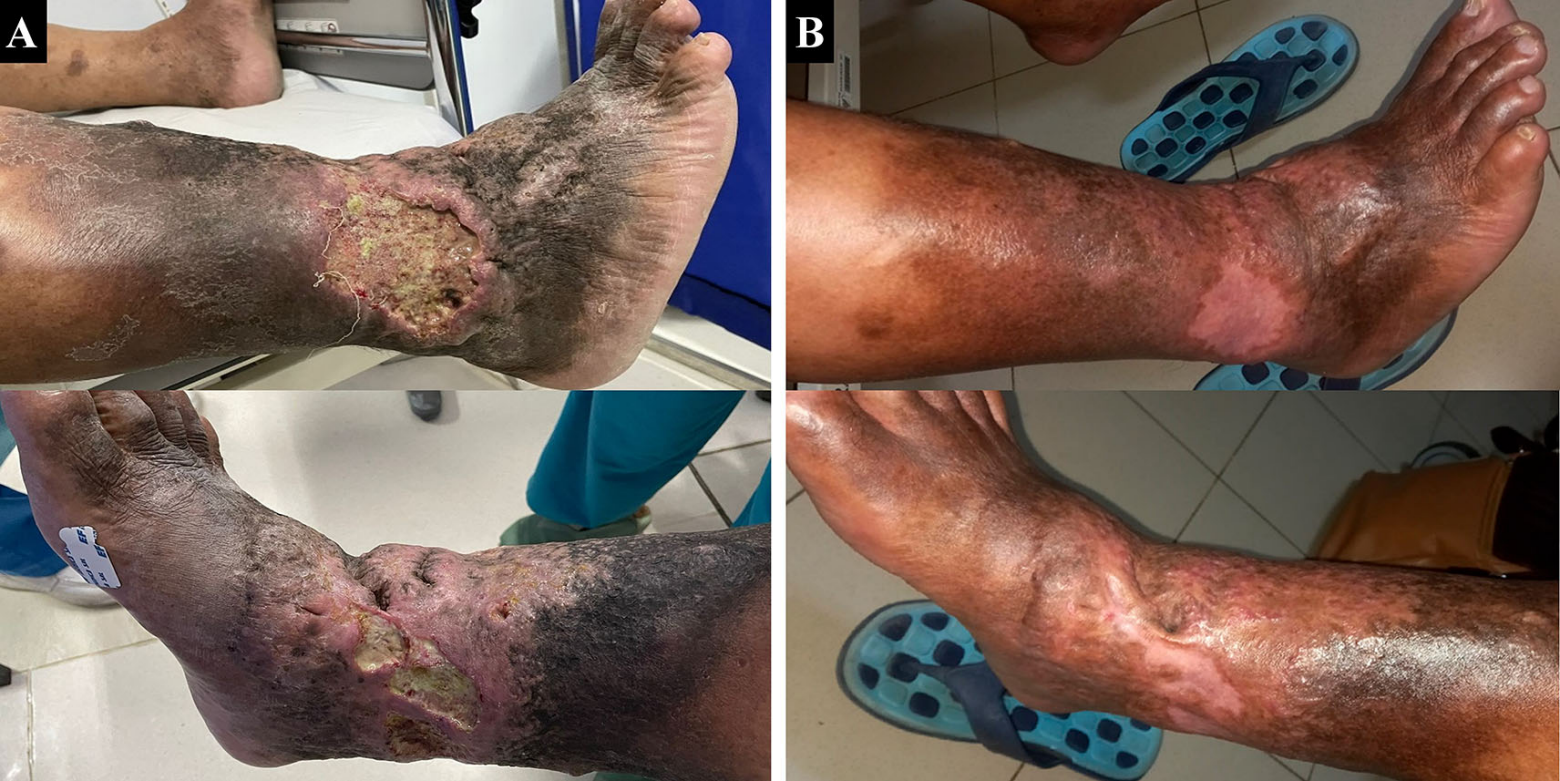
Venous leg ulcer healing progression of the 3 ^rd^ patient. A. pre-EVLA; B. 6-months post EVLA.

A 65-year-old male farmer presented with complaints of ulcers with malodorous discharge at the right ankle accompanied by pain (VAS 4/10) in the past two years which became worse and enlarged in the past six months. Complaints begin with swelling of both lower limbs for 11 years. The patient had undergone various traditional treatments before such as the use of a flour mixture. The patient had a history of hypertension and diabetes mellitus, was an ex-smoker, and was obese with a BMI of 33.3 kg/m ^2^. Physical examination showed multiple ulcers sized 10×8 cm at lateral side of the right ankle and 8×3 cm at medial side of the right ankle with irregular edge, wet base with purulent discharge, erythema, edema (+), and hyperpigmentation from 1/3 proximal of the right lower leg until dorsal side of the lower foot ( Figure 4A). Plethysmography examination revealed his ankle and arm systolic pressure, then it was divided to get the ABI with a result of his left ABI was 1.22 and right ABI was 1.01. Lower extremity DUS showed severe reflux (duration >1000 ms) of bilateral GSV, small saphenous vein (SSV), common femoral vein, and popliteal vein. No obstruction or thrombus was found in the deep vein. The flow of arteries in his both lower extremities was normal. The patient was diagnosed with bilateral CVI with active VLU at medial and lateral side of right ankle region (C6sEpAsdPr), and VCSS was 22. The patient decided to undergo EVLA on the right lower limb first and received ceftriaxone and metronidazole five days before the procedure. The procedure was done after the patient finished the antibiotics course.

EVLA was done under spinal anesthesia. Mapping was done along the right GSV and SSV. The diameter of SFJ was 12.0 mm, proximal ATK GSV was 7.5 mm, media ATK GSV was 7.0 mm, distal ATK GSV was 7.8 mm, proximal BTK GSV was 6.0 mm, media BTK GSV was 4.0 mm, and distal BTK GSV was 3.0 mm. The proximal SSV diameter was 7.0 mm, and the media SSV was 6.5 mm. Puncture by guided DUS was done at three locations for GSV: medial side of dorsalis pedis (distal from the ulcer), proximal BTK GSV with laser catheter introduced retrogradely, and proximal BTK GSV with laser catheter introduced until 3 cm passed SFJ. EVLA was done with laser power 6 W LEED 50 J/cm in proximal until media ATK GSV, 5 W LEED 40 J/cm in media ATK GSV until proximal BTK GSV, and 2 W LEED 20 J/cm in proximal until distal BTK. Puncture for SSV was done at media BTK, laser catheter was introduced until 5 cm passed Sapheno-poplitea Junction. EVLA at proximal until media SSV was done with laser power 4 W LEED 30 J/cm. The total ablated GSV length was 64 cm and the SSV length was 10 cm with a total of 850 mL of tumescent amount. Lower extremity DUS on the next day showed the ablated GSV and SSV were perfectly obliterated, no thrombus found, and a patent epigastric vein. Wound dressings of the ulcer and an elastic bandage were applied to the patient’s right lower limb.

The patient was discharged two days after the procedure with optimal medical therapy to control his risk factor and the wound care was done by the nursing team that follows the TIME concept with modern wound dressing. At follow-up until six months, the patient did not have any complaints and the ulcer and skin condition had significantly improved. There was no ulcer left, hyperpigmentation was limited to 1/3 proximal of the right lower leg, and there was a scar (+) on the previously ulcerous region ( Figure 4B). No recanalization of the treated segment was found by DUS examination. There was a 17-point reduction of VCSS down to 5.

## Discussion

According to the European Society for Vascular Surgery (ESVS) 2022 Clinical Practice Guidelines on the Management of Chronic Venous Disease of the Lower Limbs, it is recommended to do early endovenous ablation in patients presenting with active VLU and superficial vein incompetence to accelerate ulcer healing (Class 1 Recommendation with Level of Evidence). ^1^ But the topic of how to do EVLA in active VLU patients remains to be discussed.

All patients that we have reported here were classified as C6 CEAP clinical criteria with DUS examination revealing reflux of both ATK and BTK GSV, and one case each with accessory vein and SSV involvement. This clinical finding was correlated with superficial vein as mentioned in García-Gimeno *et al*.’s ^9^ study that in CVD patients, involvement of ATK and BTK GSV without SSV (odds ratio (OR) 1.72, P<0.05) or with SSV (OR 2.62, P<0.05) significantly increases the possibility of the patient to be in C4 – C6 clinical class.

Despite superficial vein reflux, VLU may also caused by deep vein pathology or even a combination of both. ^1^ Scuhl, et al. ^10^ found that in 1794 patients with a leg wound, 68.7% had isolated superficial vein pathology, 3.1% isolated deep vein pathology, 12.7% combined superficial and deep vein pathology, and 15.5% had no vein pathology. Treatment of the deep veins consists of correcting obstruction or reflux, which is performed rarely. When there is a combination of superficial and deep vein reflux, as we found in our patients, superficial reflux can be treated and sometimes may also result in segmental deep venous reflux resolution, although residual deep vein reflux itself was found not to impair the healing of VLU. ^1^ Some physicians believe that deep vein obstruction might be a contraindication for the superficial vein ablation due to potentially obliterating the collateral outflow and worsening venous hypertension. ^11^^,^^12^ However, current research showed that superficial vein reflux ablations are safe and provide benefits, even to the vein diameter and reflux in the deep vein. ^12^^–^^14^ CVD patients may experience deep venous reflux due to volume overload caused by superficial vein reflux via perforating veins, resulting in dilatation and incompetence of the deep vein valves. ^14^

Our patients were candidates for EVLA procedure since no contraindication was found including peripheral artery diseases (PAD) that were ruled out by performing an ankle-brachial index examination and lower extremity DUS examining the arteries flow although our patients have risk factors to develop PAD. We believe that significant PAD (with ABI <0.5) was a relative contraindication for EVLA that prevented the use of post-procedural compression therapy. ^1^^,^^11^ Regarding patients’ preparation before the procedure, although the current ESVS guideline ^1^ doesn’t advise routine use of systemic antimicrobials for venous leg ulcers, in our practice, we usually still use it as an empirical antibiotic in our hospital.

Numerous incidents of BTK GSV reflux after ATK GSV EVLA have been reported. A study by de Arujo *et al*. ^15^ showed a 70% incidence of BTK reflux one year after ATK GSV ablation. The study was consistent with our second case which presented persistent symptoms and delayed ulcer healing even after the treatment of ATK GSV EVLA. Pihlaja *et al*. ^16^ stated that persistent reflux of superficial vein after ablation was the main factor of delayed ulcer healing (multivariate hazard ratio (HR) 0.123, 0.0051-0.295 95% CI, P<0.001) with multifactorial etiology including missed reflux segment and incomplete therapy, reflux sources (GSV/SSV/perforator) which were normal at the earlier examination, recanalization of the treated segment, and history of deep vein thrombosis.

The decision to do GSV EVLA until BTK is preferred because it is supported by numerous pieces of evidence that provide significantly more advantages than only ATK GSV EVLA. Elimination of reflux throughout the length of the incompetent GSV segment from the SFJ to the most distal point will lead to significant clinical improvement and reduce the need for adjuvant therapy. ^15^ A systematic review by Sussman *et al*. ^17^ mentioned that in CVI with C4 to C6 disease, more aggressive treatment of the ATK and BTK GSV is justified if the DUS findings demonstrate groin to ankle reflux. These pieces of evidence support our decision to treat our patient with EVLA until BTK GSV where there is still significant reflux.

In comparison to foam sclerotherapy, numerous studies showed that foam sclerotherapy has a higher recurrence rate compared to EVLA, ^18^^–^^20^ so in our case, foam sclerotherapy is not an option that we would choose.

However, there is another factor that should be taken into consideration which is the risk of complications. Reported complications of EVLA procedure are infection (0.5%), thromboembolic events such as deep vein thrombosis and endovenous heat-induced thrombosis (0.85%), hematoma (2.1%), skin burn (2.1%), short term paraesthesia (3.8%), superficial thrombophlebitis (5.6%), and bruising (39%). ^21^ Nerve injury risk in EVLA is directly related to the distance of the ablated vein with the nerve through the needle stick mechanism or burn due to laser energy transfer. This means that BTK GSV EVLA had a higher risk of causing nerve injury since the saphenous nerve runs medially towards the GSV at the level of the upper calf to the ankle. ^22^ Sinabulya *et al*. ^23^ demonstrated a non-clinically significant permanent sensory loss at 8% of limbs that underwent BTK GSV EVLA after a mean of 41 months follow-up duration in VLU patients without any major nerve injury reported. Utoh and Tsukamoto observed the effect of ablation length on the incidence of post-EVLA neuropathy with an incidence of 0.8% if the ablation length was less than 40 cm from the SFJ and 4.6% if it was more or equal to 40 cm. ^24^ All of the patients in our cases did not show any nerve injury symptoms after follow-up until six months.

Various efforts need to be made to minimize complications from EVLA. Tumescent anesthesia is used in EVLA to prevent thermal-induced tissue damage, compressing the size of the vein to be treated to allow better energy absorption, as well as allowing early mobilization and reducing the risk of DVT where EVLA without tumescence has an incidence of approximately 36.5% of nerve-related paresthesia. ^22^^,^^25^^,^^26^ Reducing the number of punctures while administering tumescent anesthesia also logically reduces the incidence of nerve injury. ^15^ We administered DUS-guided tumescent anesthesia in our patients and at follow-up, our patients did not show any signs or symptoms of nerve injury.

We believe these cases can be done with tumescence anesthesia alone, but spinal anesthesia showed significantly lower pain scores compared to tumescence alone. ^27^ Our patients already experienced pain related to their VLU so we assumed it would be better to minimize their pain-related procedure with the choice of spinal anesthesia despite the longer preparation time and spinal anesthesia-related complications which fortunately didn’t happen in our patients.

It’s hard to determine the cut-off of laser power in EVLA to perfectly obliterate the vein without complication. Reducing the laser energy along the distal segment of BTK GSV may reduce the incidence of paresthesia but with consequences of decreased treatment success. ^15^ The newer generation of EVLA with 1470-nm wavelength has more advantages with chromophore targeting only water in the endothelium compared to earlier laser generations with 940-nm and 980-nm wavelengths that targeted hemoglobin molecules. ^28^^,^^29^ EVLA with higher wavelength is related to lower energy levels and minimal side effects. ^29^ A study by Arslan *et al*. ^29^ showed recanalization numbers were significantly lower (P=0.017) after 48 months of follow-up in a group of EVLA with 1470-nm radial-tip laser catheters using laser power LEED 45–120 J/cm (8.3%) compared to the group with 980-nm bare-tip laser catheter using laser power LEED 60–120 J/cm (15.9%). Post-procedural complications such as pain, induration, ecchymosis, paresthesia, and DVT were significantly lower in the 1470-nm group. In another study by Doganci and Demirkilic, 3.33% of the 1470-nm group reported paresthesia compared to 30% in the 980-nm group (P<0.0001). The symptoms experienced tended to be minor with an average duration of less than four weeks in both groups. ^30^

Although a systematic review and meta-analysis by Malskat *et al*. ^31^ showed that the commonly used EVLA parameters (wavelength, delivered energy, and outcome) did not affect the high success rate of the therapy (92%), the laser energy used in this study has a cut off of 50 J/cm. Mendes-Pinto *et al.* ^32^ showed that closure rates at one-year follow-up were lower in a group of 1920-nm EVLA with LEED 17.8±0.6 J/cm (87.5%) compared to the 1470-nm group with LEED 24.7±0.8 J/cm (94.7%) whereas Park *et al*. ^28^ performed EVLA using a 1470-nm laser with laser energy 6W LEED 30 J/cm on 267 limbs and obtained 84.5% completely obliterated GSV results in one month and 100% after six months. Utoh and Tsukamoto proposed EVLA with two-step ablation protocol in their trial that involved 90 limbs, using a 1470-nm laser with energy 7W in ATK GSV (LEED 50 – 70 J/cm) and 5W in BTK GSV (LEED 20-25 J/cm) with mean ablated GSV length of 50.4 cm. The DUS follow-up resulted in perfect obliteration of the ablated vein without any saphenous nerve injuries. ^33^ In our case, we were using a protocol with lower laser energy than some past literature because of anatomical consideration that the average GSV diameter of an Asian population from a Singaporean study was significantly lower than a Caucasian population (Median 2.9 mm vs 5.7 mm; P<0.01) although this result can be biased since those Caucasian patients may be presented earlier. ^34^ Indeed, further studies with the use of low EVLA energy with larger sample and longer follow-up period should be made in the future.

Post-procedural management of EVLA also has an important role in giving better outcomes for the patients. We perform clinical follow-up and lower extremity DUS one day after the procedure to assess the treatment efficacy, possible complications, and excluding thrombosis. Thereby, in our cases, the patients were hospitalized with another consideration that the patients traveled quite far away to our center so it would be harder to monitor the patient’s post-procedural condition. Although we believe that EVLA can be done in outpatient settings in certain conditions. After that, routine DUS follow-up was performed within 3–6 months after the procedure, according to the Guidelines of the First International Consensus Conference on Endovenous Thermal Ablation for Varicose Vein Disease. ^11^

Our patients are also discharged with optimal medical treatment to control their risk factors adequately and referred to the nursing team that specializes in wound care. Wound care was done by following the TIME concept that was proposed by Schultz *et al.* ^35^ with a combination use of modern wound dressing. The tissue component provides viable wound tissue by debridement. In our case, we used a combination of chemical, autolytic, and sharp debridement. The debridement process was done until granulation tissue was formed in approximately less than 2 weeks. The infection/inflammation control was done with combination use of cadexomer iodine, octenidine dihydro-chloride, polyhexamethylenebiguanide, and concentrated surfactant gels. Moisture should be balanced, and, in our case, we reduce the excessive fluid that causes maceration of the wound margin by using calcium alginate and hydrofoam. The edge of the wound was assessed with the target of advancing epidermal margin and decreased wound stage. The wounds were then covered with transparent film and collagen dressing. Another important component is the use of a 3-layer tubular compression bandage to achieve 40–50 mmHg pressure which is known to be useful in promoting VLU healing. ^1^^,^^36^

The limitations of current case reports are lack of follow up duration and reduced number of patients included. Further studies with a larger sample size and longer follow-up duration should be conducted in the future.

## Conclusion

We’ve reported three cases of patients with active VLU undergoing EVLA until BTK with significant results. The EVLA of GSV until BTK where there is still significant reflux provides satisfactory results in patients with VLU. A combination of DUS-guided procedure, administration of tumescent anesthesia, use of newer generation devices, minimal laser energy, and post-procedural management could minimize the risk of complications and produce a maximal outcome.

## Research ethics and patient consent

Written informed consent has been obtained from the patients for publication of the case report and accompanying images.

## Data Availability

All data underlying the results are available as part of the article and no additional source data are required.
