# Supplementary material for: Satisfactory result of great saphenous vein endovenous laser ablation until below the knee on active venous leg ulcer: a case series
Source: F1000Res. 2024 Sep 20;12:383. Originally published 2023 Apr 12. [Version 4] doi: 10.12688/f1000research.131695.4 (PMC11377932; doi:10.12688/f1000research.131695.4)
Supplement: Peer review history

# Reviewer report: Reviewer response for version 4

**DOI:** 10.5256/f1000research.171959.r325501

**Published:** 2024-09-27

**Authors:** Marina Dias-Neto

**Affiliations:** 1 Department of Cardiothoracic and Vascular Surgery, The University of Texas Health Science Centre at Houston, Houston, USA; 2 Angiologia e Cirurgia Vascular, Unidade de Saúde Local de São João, Porto, Portugal; 3 University of Porto, Porto, Portugal

The authors addressed the all the topics suggested. I have not further comments.

Are enough details provided of any physical examination and diagnostic tests, treatment given and outcomes?

Yes

Is the background of the cases’ history and progression described in sufficient detail?

Yes

Is sufficient discussion included of the importance of the findings and their relevance to future understanding of disease processes, diagnosis or treatment?

Yes

Is the conclusion balanced and justified on the basis of the findings?

Yes

Reviewer Expertise:

MD PhD in Cardiovascular sciences. Areas of interest: aortic aneurysm, peripheral artery disease, venous disease.

I confirm that I have read this submission and believe that I have an appropriate level of expertise to confirm that it is of an acceptable scientific standard.

# Reviewer report: Reviewer response for version 4

**DOI:** 10.5256/f1000research.171959.r325505

**Published:** 2024-09-25

**Authors:** Patrizia Pavei

**Affiliations:** 1 Department of Surgery, University Hospital of Padua, Padua, Italy

Approved

Are enough details provided of any physical examination and diagnostic tests, treatment given and outcomes?

Partly

Is the background of the cases’ history and progression described in sufficient detail?

Yes

Is sufficient discussion included of the importance of the findings and their relevance to future understanding of disease processes, diagnosis or treatment?

Partly

Is the conclusion balanced and justified on the basis of the findings?

Yes

Reviewer Expertise:

Phlebology; endovenous treatment of varicose veins (Laser Radiofrequency, glue, UGFS), treatment of venous ulcers

I confirm that I have read this submission and believe that I have an appropriate level of expertise to confirm that it is of an acceptable scientific standard.

# Reviewer report: Reviewer response for version 3

**DOI:** 10.5256/f1000research.163154.r323482

**Published:** 2024-09-14

**Authors:** Muhamad Taufik Ismail

**Affiliations:** 1 Sardjito Central General Hospital/Faculty of Medicine, Public Health, and Nursing, Gadjah Mada University,, Yogyakarta, Indonesia

These case series are well-detailed and offer comprehensive information on the patient's illustration from history, and diagnosis, to treatment approach. However, based on the CARE guidelines for writing case series, it is important to address several key components, including limitations in patient management and obtaining informed consent from patients. This provided case reports lack explicit mention of these aspects.

Are enough details provided of any physical examination and diagnostic tests, treatment given and outcomes?

Yes

Is the background of the cases’ history and progression described in sufficient detail?

Yes

Is sufficient discussion included of the importance of the findings and their relevance to future understanding of disease processes, diagnosis or treatment?

Yes

Is the conclusion balanced and justified on the basis of the findings?

Yes

Reviewer Expertise:

NA

I confirm that I have read this submission and believe that I have an appropriate level of expertise to confirm that it is of an acceptable scientific standard.

# Response

**Published:** 2024-09-15

**Authors:** Taofan Taofan

**Affiliations:** National Cardiovascular Center Harapan Kita, Indonesia

Thank you for your humble review regarding our article. Here are our responses based on your review:

**Reviewer Comment:**

**...based on the CARE guidelines for writing case series, it is important to address several key components, including limitations in patient management and obtaining informed consent from patients**

**Author Response:**

 We’ve mentioned some of the limitations in patient management implicitly such as routine use of systemic antimicrobials and the choice of spinal anesthesia in our patients. We’ve also stated that written informed consent has been obtained from the patients for publication of the case report and accompanying images.

 Hope that our responses can clarify some of the confusion. Once again, thank you Dr. Muhamad Taufik Ismail for the insights regarding our manuscript. Please let us know if further revision or information is needed.

 Best Regards,

 Taofan

 Department of Cardiology and Vascular Medicine, Faculty of Medicine University of Indonesia / National Cardiovascular Center Harapan Kita / University of Indonesia Academic Hospital, Jakarta, Indonesia

# Reviewer report: Reviewer response for version 3

**DOI:** 10.5256/f1000research.163154.r304555

**Published:** 2024-09-05

**Authors:** Marina Dias-Neto

**Affiliations:** 1 Department of Cardiothoracic and Vascular Surgery, The University of Texas Health Science Centre at Houston, Houston, USA; 2 Angiologia e Cirurgia Vascular, Unidade de Saúde Local de São João, Porto, Portugal; 3 University of Porto, Porto, Portugal

**This is a very interesting paper about **great saphenous vein endovenous laser ablation until below the knee on active venous leg ulcer. The authors report 3 cases that are well documented regarding physical examination, diagnostic tests, treatment given and outcomes.

**I believe the paper reads well and will be useful for readers that treat this type of patients.**

 I have only minor comments:

 The authors wrote that “plethysmography examination revealed (…) ankle-brachial index (ABI)” > I wonder how do the authors used Plethysmography to determine ABI? Maybe this should be corrected since ABI is not an outcome of Plethysmography but of a arterial pressure assessment.

 In case 2, the presence of axial deep venous reflux might be a relative contra-indication to treat the superficial venous system, since it is acting as a compensatory pathway to deep venous disease. I would like the authors to explain a bit better what they thought otherwise.

 Finally, the main criticism to the cases could be a lack of a longer follow up. This could be acknowledged in a paragraph highlighting the limitations of the study. Another limitation is the reduced number of patients included.

Are enough details provided of any physical examination and diagnostic tests, treatment given and outcomes?

Yes

Is the background of the cases’ history and progression described in sufficient detail?

Yes

Is sufficient discussion included of the importance of the findings and their relevance to future understanding of disease processes, diagnosis or treatment?

Yes

Is the conclusion balanced and justified on the basis of the findings?

Yes

Reviewer Expertise:

MD PhD in Cardiovascular sciences. Areas of interest: aortic aneurysm, peripheral artery disease, venous disease.

I confirm that I have read this submission and believe that I have an appropriate level of expertise to confirm that it is of an acceptable scientific standard, however I have significant reservations, as outlined above.

# Response

**Published:** 2024-09-15

**Authors:** Taofan Taofan

**Affiliations:** National Cardiovascular Center Harapan Kita, Indonesia

We appreciate your constructive feedback on our article. Below, we address each of the comments provided.

**The authors wrote that “plethysmography examination revealed (…) ankle-brachial index (ABI)” > I wonder how do the authors used Plethysmography to determine ABI? Maybe this should be corrected since ABI is not an outcome of Plethysmography but of a arterial pressure assessment.**

 We would like to clarify that plethysmography, specifically air plethysmography, can provide indirect information that assists in the calculation of ABI by measuring volume changes in the limbs during a pressure assessment. While Doppler ultrasound is indeed the most common method for directly measuring ABI, our statement was intended to reflect a comprehensive evaluation where plethysmography was part of the vascular assessment that included ABI measurement.

 In clinical practice, combining plethysmography with other assessments, including Doppler, can provide a more complete picture of both arterial and venous circulation. However, we acknowledge that the wording in the manuscript may have led to confusion, implying that plethysmography alone was used to determine ABI. We have revised the text to clarify that ABI was primarily determined using Doppler ultrasound and blood pressure cuffs, with plethysmography used to assess complementary parameters such as venous reflux.

**In case 2, the presence of axial deep venous reflux might be a relative contra-indication to treat the superficial venous system, since it is acting as a compensatory pathway to deep venous disease. I would like the authors to explain a bit better what they thought otherwise.**

 In response to the reviewer's suggestion regarding the presence of axial deep venous reflux in Case 2, we agree that it may act as a compensatory pathway to deep venous disease. We recognize that treating the superficial venous system without addressing the deep venous reflux could potentially compromise this compensatory mechanism. However, in our case, the patient had persistent symptoms and a non-healing ulcer despite previous above-the-knee EVLA, suggesting that the superficial reflux was a significant contributing factor to the disease state. We hypothesized that treating the superficial reflux could help manage the venous hypertension, while careful follow-up would be essential to monitor for any adverse effects related to the deep system. We have expanded this discussion in the manuscript to clarify our rationale.

**Finally, the main criticism to the cases could be a lack of a longer follow up. This could be acknowledged in a paragraph highlighting the limitations of the study. Another limitation is the reduced number of patients included.**

 We agree with the reviewer that the limited number of cases and relatively short follow-up period are significant limitations of our study. We have added a new paragraph in the discussion section to address these points. Specifically, we acknowledge that the small sample size limits the generalizability of our findings, and the follow-up duration of six months may not fully capture late recurrences or complications such as recanalization. Future studies with a larger cohort and longer follow-up period are needed to validate our findings further.

 Once again, thank you for the humble insights regarding our manuscript from Dr. Marina Dias-Neto. Hope our answers and revision can complete our manuscript and please let us know if further revision is needed.

 Sincerely,

 Taofan

 Department of Cardiology and Vascular Medicine, Faculty of Medicine University of Indonesia / National Cardiovascular Center Harapan Kita / University of Indonesia Academic Hospital, Jakarta, Indonesia

# Reviewer report: Reviewer response for version 3

**DOI:** 10.5256/f1000research.163154.r251651

**Published:** 2024-03-05

**Authors:** Patrizia Pavei

**Affiliations:** 1 Department of Surgery, University Hospital of Padua, Padua, Italy

The authors have addressed all the concerns I raised in my review report appropriately.

Are enough details provided of any physical examination and diagnostic tests, treatment given and outcomes?

Partly

Is the background of the cases’ history and progression described in sufficient detail?

Yes

Is sufficient discussion included of the importance of the findings and their relevance to future understanding of disease processes, diagnosis or treatment?

Partly

Is the conclusion balanced and justified on the basis of the findings?

Yes

Reviewer Expertise:

Phlebology; endovenous treatment of varicose veins (Laser Radiofrequency, glue, UGFS), treatment of venous ulcers

I confirm that I have read this submission and believe that I have an appropriate level of expertise to confirm that it is of an acceptable scientific standard.

# Reviewer report: Reviewer response for version 2

**DOI:** 10.5256/f1000research.158206.r224117

**Published:** 2024-02-21

**Authors:** Patrizia Pavei

**Affiliations:** 1 Department of Surgery, University Hospital of Padua, Padua, Italy

Patrizia Pavei

 Day Surgery Week Surgery Multidisciplinare

 Azienda Ospedale Università Padua

 Padua - ITALY

 This paper reports on three impressive cases of active venous ulcers of the lower limb treated with endovenous laser ablation.

 The advantage of early treatment with endovenous thermal ablation in patients with an active venous ulcer from superficial venous insufficiency has been described in the literature.

 These three case reports prove that this approach is equally efficacious in complicated cases.

 Some considerations:

 Italian terms have been used (for example *sinistra* and *destra*). Perhaps the English words would be preferable.

 In all of the three cases, a “moderate reflux” or “ reflux “ in the deep veins is described.

 Which deep veins were involved? And what do the authors mean by “moderate reflux”? Were these patients studied with Plethysmography? Did these cases have a post-thrombotic syndrome?

 The presence of  primary or secondary deep vein insufficiency may be responsible for recurrent ulcers. Could the authors comment on this in the discussion?

 The use of very low energy ( 2W; LEED 20J/cm) in the BTK trunk could be a cause of recanalization over time.

 This point should be discussed even if the primary endpoint of faster VLU healing has been obtained.

Are enough details provided of any physical examination and diagnostic tests, treatment given and outcomes?

Partly

Is the background of the cases’ history and progression described in sufficient detail?

Yes

Is sufficient discussion included of the importance of the findings and their relevance to future understanding of disease processes, diagnosis or treatment?

Partly

Is the conclusion balanced and justified on the basis of the findings?

Yes

Reviewer Expertise:

Phlebology; endovenous treatment of varicose veins (Laser Radiofrequency, glue, UGFS), treatment of venous ulcers

I confirm that I have read this submission and believe that I have an appropriate level of expertise to confirm that it is of an acceptable scientific standard, however I have significant reservations, as outlined above.

# Response

**Published:** 2024-02-26

**Authors:** Taofan Taofan

**Affiliations:** National Cardiovascular Center Harapan Kita, Indonesia

Thank you so much for the wonderful review of our manuscript. Here we try to answer several of the comments.

**Italian terms have been used (for example sinistra and destra). Perhaps the English words would be preferable.**

 Thank you for the insights, we’ve changed the terms into English.

**In all of the three cases, a “moderate reflux” or “reflux “in the deep veins is described.**

**Which deep veins were involved?**

 Common femoral vein and popliteal vein. We’ve changed the deep vein term in the manuscript.

**And what do the authors mean by “moderate reflux”?**

 Moderate reflux means the duration of the reflux is between 500 ms – 1000 ms. We’ve added the explanation in the manuscript.

**Were these patients studied with Plethysmography?**

 Yes, the ankle-brachial index in our patients was obtained using plethysmography. We’ve re-mentioned it in the manuscript.

**Did these cases have post-thrombotic syndrome?**

 No post-thrombotic syndrome, DVT, or E-HIT was found in our patients before the procedure and until 6 months follow-up. Our patients undergo duplex ultrasound examination before the procedure, 1 day, 1 month, 3 months, and 6 months after the procedure.

**The presence of primary or secondary deep vein insufficiency may be responsible for recurrent ulcers. Could the authors comment on this in the discussion?**

 We’ve added this topic to the discussion:

 “Despite superficial vein reflux, VLU may also caused by deep vein pathology or even a combination of both. Scuhl, et al. found that in 1794 patients with a leg wound, 68.7% had isolated superficial vein pathology, 3.1% isolated deep vein pathology, 12.7% combined superficial and deep vein pathology, and 15.5% had no vein pathology. Treatment of the deep veins consists of correcting obstruction or reflux, which is performed rarely. When there is a combination of superficial and deep vein reflux, as we found in our patients, superficial reflux can be treated and sometimes may also result in segmental deep venous reflux resolution, although residual deep vein reflux itself was found not to impair the healing of VLU.”

**The use of very low energy (2W; LEED 20J/cm) in the BTK trunk could be a cause of recanalization over time. This point should be discussed even if the primary endpoint of faster VLU healing has been obtained.**

 We believe that the use of low EVLA energy in our cases should be sufficient to prevent recanalization over time for a few reasons:

1. Anatomical consideration that the average GSV diameter of an Asian population was significantly lower than a Caucasian population.
2. A study by Utoh and Tsukamoto with a two-step ablation protocol using a 1470-nm laser with energy 7W in ATK GSV (LEED 50 – 70 J/cm) and 5W in BTK GSV (LEED 20-25 J/cm) was found to be a safe and efficient procedure.
3. We’ve done routine DUS follow-ups in our patients to screen the possibility of recanalization over time.
4. Indeed, further studies should be made regarding the use of low EVLA energy with larger samples and longer follow-up periods.

Once again, thank you for the humble insights regarding our manuscript from Dr. Patrizia Pavei. Hope our answers and revision can complete our manuscript and please let us know if further revision is needed.

# Reviewer report: Reviewer response for version 1

**DOI:** 10.5256/f1000research.144563.r202935

**Published:** 2023-10-24

**Authors:** Windsor Ting

**Affiliations:** 1 Division of Vascular Surgery, Department of Surgery, Icahn School of Medicine at Mount Sinai, New York, USA

This manuscript reports on three cases of active venous ulcers from superficial venous insufficiency treated with endovenous laser ablation.  

 The efficacy and safety of endovenous thermal ablation in patients with an active venous ulcer from superficial venous insufficiency have been reported. Nevertheless, there is an important role for case reports of these patients because it provides a picture of these patients in the real world.

 This manuscript is overall well-written and potentially suitable for indexing. 

 Several comments:

 There are numerous grammatical errors in the manuscript. The authors should try to correct these errors before indexing of the manuscript. 

 The term "chronic venous insufficiency (CVI)" is used throughout the manuscript. CVI means different things to different people. Please define CVI so it is clear what exactly is CVI.

 "Treatment of superficial venous reflux with early endovenous laser ablation (EVLA) has been proven to reduce ulcer recurrence levels and promote faster VLU healing."

 It has been shown or associated with. "Prove" is too strong a statement. 

 "and received ceftriaxone antibiotics for five days"

 Why antibiotic? Did the patient have a positive wound culture? Does the author use antibiotic routinely in these cases?

 "EVLA was performed under spinal anesthesia." 

 Why spinal anesthesia? These cases can be done with with just tumescence. 

 "Lower extremity DUS showed perfectly obliterated ablated GSV, no thrombus found in the deep vein, and a patent epigastric vein."

 When was this performed? What is the authors' recommendation regarding postoperative ultrasound?

 "The patient was discharged one day after the procedure."

 Why was the patient hospitalized?

 "The patient had a history of hypertension, type 2 diabetes mellitus, and obesity with body mass index (BMI) of 39.45 kg/m ^2^."

 Did any of these patients have peripheral artery disease? At least one patient was a smoker. How was PAD ruled out? Is it necessary to rule out PAD?

 "The patient was discharged two days after the procedure with optimal medical treatment and wound care by the nursing team with modern wound dressing."

 What is optimal medical treatment?

 What did the wound care consist of?

 What is modern wound dressing?

 "smelly discharge"

 Malodorous discharge is a better medical term. 

 "The decision to do GSV EVLA until BTK is preferred because it is supported by numerous pieces of evidence that provide significantly more advantages than only ATK GSV EVLA."

 How far below the knee? 

 "Various efforts need to be made to minimize complications from EVLA. Tumescent anesthesia is used in EVLA to prevent thermal-induced tissue damage,"

 What about foam sclerotherapy for the GSV below the knee?

 "the average GSV diameter of an Asian population was significantly lower than a Caucasian population (Median 2.9 mm vs 5.7 mm; P<0.01). ^25"^

 Is that really true? GSV reflux is diagnosed much earlier in Caucasian population whereas in the Asian population, saphenous vein reflux is usually diagnosed late as demonstrated in your three cases.

Are enough details provided of any physical examination and diagnostic tests, treatment given and outcomes?

Yes

Is the background of the cases’ history and progression described in sufficient detail?

Yes

Is sufficient discussion included of the importance of the findings and their relevance to future understanding of disease processes, diagnosis or treatment?

Yes

Is the conclusion balanced and justified on the basis of the findings?

Yes

Reviewer Expertise:

Venous disease

I confirm that I have read this submission and believe that I have an appropriate level of expertise to confirm that it is of an acceptable scientific standard, however I have significant reservations, as outlined above.

# Response

**Published:** 2023-11-15

**Authors:** Taofan Taofan

**Affiliations:** National Cardiovascular Center Harapan Kita, Indonesia

Thank you very much for the wonderful insights from Dr. Windsor Ting regarding our manuscript. He gave us constructive comments to improve our manuscript. Here we will try to answer several comments from him.

**1. There are numerous grammatical errors in the manuscript. The authors should try to correct these errors before indexing the manuscript.**

 Thank you for the comments, we try our best to revise the grammatical errors in our manuscript.

**2. The term "chronic venous insufficiency (CVI)" is used throughout the manuscript. CVI means different things to different people. Please define CVI so it is clear what exactly is CVI.**

 Chronic venous disease (CVD) is defined as a long-term morphological and functional abnormality of the venous system that manifests as symptoms or signs requiring treatment or examination. Chronic venous insufficiency (CVI), which correlates with clinical, etiological, anatomical, and pathophysiological (CEAP) classification as C3 through C6, is reserved for advanced CVD and applied to functional anomalies of the venous system that result in edema, skin changes, or venous ulcers.

**3. "Treatment of superficial venous reflux with early endovenous laser ablation (EVLA) has been proven to reduce ulcer recurrence levels and promote faster VLU healing."** **It has been shown or associated with. "Prove" is too strong a statement.**

 Thank you for the advice. We have changed the statement to “Treatment of superficial venous reflux with early endovenous laser ablation (EVLA) has been associated with reducing ulcer recurrence levels and promoting faster VLU healing.”

**4. "and received ceftriaxone antibiotics for five days"** **Why antibiotic? Did the patient have a positive wound culture? Does the author use antibiotic routinely in these cases?**

 Although the current ESVS chronic venous disease guideline doesn’t advise routine use of systemic antimicrobials for venous leg ulcers, in our practice, we usually still use it as an empirical antibiotic in our hospital.

**5. "EVLA was performed under spinal anesthesia."** **Why spinal anesthesia? These cases can be done with just tumescence.**

 We believe these cases can be done with tumescence anesthesia alone, but spinal anesthesia showed significantly lower pain scores compared to tumescence alone. Our patients already experienced pain related to their VLU so we assumed it would be better to minimize their pain-related procedure with the choice of spinal anesthesia despite the longer preparation time and spinal anesthesia-related complications which fortunately didn’t happen in our patients. For that reason, we also prefer to hospitalize our patients.

**6. “Lower extremity DUS showed perfectly obliterated ablated GSV, no thrombus found in the deep vein, and a patent epigastric vein.”** **When was this performed? What is the authors’ recommendation regarding postoperative ultrasound?**

 The lower extremity DUS was performed one day after the procedure. After that, routine DUS follow-up was performed within 3 – 6 months after the procedure, according to the Guidelines of the First International Consensus Conference on Endovenous Thermal Ablation for Varicose Vein Disease.

**7. “The patient was discharged one day after the procedure. ”** **Why was the patient hospitalized?**

 The patient was hospitalized to perform the post-procedural lower extremity DUS. Another consideration was the patient traveled quite far away to our center so it would be harder to monitor the patient’s post-procedural condition. Although we believe that EVLA can be done in outpatient settings in certain conditions.

**8. “The patient had a history of hypertension, type 2 diabetes mellitus, and obesity with body mass index (BMI) of 39.45 kg/m2.”** **Did any of these patients have peripheral artery disease? At least one patient was a smoker. How was PAD ruled out? Is it necessary to rule out PAD?**

 We ruled out PAD by performing an ankle-brachial index examination and lower extremity DUS examining the arteries flow. We believe that significant PAD (with ABI <0.5) was a relative contraindication for EVLA that prevented the use of post-procedural compression therapy.

**9. “The patient was discharged two days after the procedure with optimal medical treatment and wound care by the nursing team with modern wound dressing.”**

**What is optimal medical treatment?**

 Optimal medical treatment here means adequate medical treatment to control our patient’s risk factors.

**What did the wound care consist of? What is modern wound dressing?**

 Wound care was done by following the TIME concept that was proposed by Schultz et al. ^4^ with a combination use of modern wound dressing. The tissue component provides viable wound tissue by debridement. In our case, we used a combination of chemical, autolytic, and sharp debridement. The debridement process was done until granulation tissue was formed in approximately less than 2 weeks. The infection/inflammation control was done with combination use of cadexomer iodine, octenidine dihydro-chloride, polyhexamethylenebiguanide, and concentrated surfactant gels. Moisture should be balanced, and, in our case, we reduce the excessive fluid that causes maceration of the wound margin by using calcium alginate and hydrofoam. The edge of the wound was assessed with the target of advancing epidermal margin and decreased wound stage. The wounds were then covered with transparent film and collagen dressing. Another important component is the use of a 3-layer tubular compression bandage to achieve 40 – 50 mmHg pressure which is known to be useful in promoting VLU healing.

**10. "smelly discharge"** **Malodorous discharge is a better medical term.**

 Thank you for the advice, we have changed this term.

**11. "The decision to do GSV EVLA until BTK is preferred because it is supported by numerous pieces of evidence that provide significantly more advantages than only ATK GSV EVLA."** **How far below the knee?**

 Until the lowest part where there is still significant reflux.

**12. "Various efforts need to be made to minimize complications from EVLA. Tumescent anesthesia is used in EVLA to prevent thermal-induced tissue damage, "** **What about foam sclerotherapy for the GSV below the knee?**

 We believe that foam sclerotherapy has a higher recurrence rate, so in our cases, foam sclerotherapy is not an option. ^6–8^

**13. "the average GSV diameter of an Asian population was significantly lower than a Caucasian population (Median 2.9 mm vs 5.7 mm; P<0.01).25"** **Is that really true? GSV reflux is diagnosed much earlier in Caucasian population whereas in the Asian population, saphenous vein reflux is usually diagnosed late as demonstrated in your three cases.**

 We and the authors of the article believe that the result can be biased since those Caucasian patients may have been presented earlier. But we can’t conclude that the result didn’t represent the actual condition since 1) Singapore is a highly developed country so we believe that the diagnosis that was made won’t be too late. 2) If the diagnosis was made later, we believe that the GSV diameter of the Asian population would be larger than the Caucasian population that was diagnosed earlier.

 Once again, we would like to thank you for the opportunity for our manuscript to be reviewed by such an experienced doctor in the field. Hope our answers and revision can satisfy him and please let us know if further revision is needed.
